# Dr. Jekyll and Mr. Hyde? Physiology and Pathology of Neuronal Stress Granules

**DOI:** 10.3389/fcell.2021.609698

**Published:** 2021-02-25

**Authors:** Pureum Jeon, Jin A. Lee

**Affiliations:** Department of Biotechnology and Biological Sciences, Hannam University, Daejeon, South Korea

**Keywords:** stress granule, RNA granule, RNA binding protein, neurodegenerative disease, membraneless organelle

## Abstract

Stress granules (SGs) are membraneless cytosolic granules containing dense aggregations of RNA-binding proteins and RNAs. They appear in the cytosol under stress conditions and inhibit the initiation of mRNA translation. SGs are dynamically assembled under stressful conditions and rapidly disassembled after stress removal. They are heterogeneous in their RNA and protein content and are cell type- and stress-specific. In post-mitotic neurons, which do not divide, the dynamics of neuronal SGs are tightly regulated, implying that their dysregulation leads to neurodegeneration. Mutations in RNA-binding proteins are associated with SGs. SG components accumulate in cytosolic inclusions in many neurodegenerative diseases, such as frontotemporal dementia and amyotrophic lateral sclerosis. Although SGs primarily mediate a pro-survival adaptive response to cellular stress, abnormal persistent SGs might develop into aggregates and link to the pathogenesis of diseases. In this review, we present recent advances in the study of neuronal SGs in physiology and pathology, and discuss potential therapeutic approaches to remove abnormal, persistent SGs associated with neurodegeneration.

## Introduction

Neurons include unique structures, such as axons and dendrites, which are membrane-bound organelles with specialized functions. However, recent studies on membraneless compartments have found them to be important in neurons, which use dynamic cellular signaling (Ryan and Fawzi, 2019; Wolozin and Ivanov, 2019). There are many membraneless organelles in the nucleus and cytoplasm, including the nucleolus and paraspeckles or SGs, RNA transport granules, and P-bodies. Their diversity implies a diversity of biological functions, as do their distinct composition of proteins and nucleic acids (Lafarga et al., 2017; Formicola et al., 2019; Ryan and Fawzi, 2019).

Membraneless organelles are dynamically assembled via liquid-liquid phase separation (LLPS) and self-assembly of proteins or nucleic acids into different liquid phases (Wu et al., 2020). Ribonucleoprotein (RNP) granules that are composed of hundreds of RNAs and proteins are membraneless organelles (Han et al., 2012; Mittag and Parker, 2018). Very interestingly, intrinsically disordered regions (IDRs) or RNA-binding domains containing proteins existed in RNP granules (Webber et al., 2020). There are at least two types of IDRs: one is enriched in a specific amino acid sequence containing motifs, called low complexity domains (LCDs), while the others are classified as prion-like domains (PLDs) (van der Lee et al., 2014; Babinchak and Surewicz, 2020).

Intriguingly, disease-causing mutations in several neurodegenerative diseases are found within the IDRs of many granule-associated proteins (Babinchak and Surewicz, 2020). IDRs could lead to an accelerated fusion of SGs or aggregate-prone proteins, which might develop into persistent protein aggregates or inclusions (Protter et al., 2018).

Whether or how physiological granules become persistent granules develop into pathogenic aggregates *in vivo*, and how this process occurs at the cellular or molecular levels remains unclear. Little is known about how we can selectively remove these granules when treating neurodegenerative diseases.

In this review, we summarize components of neuronal stress granules (SGs) crucial for neural function and focus on describing the physiology and pathology of neuronal SGs. Finally, we discuss therapeutic approaches to treat neurodegenerative diseases associated with pathological SGs.

## Stress Granules

Neuronal RNA granules are dense cytoplasmic RNA granules associated with mRNA metabolism in cell bodies, axons, and dendrites of developing and mature neurons (Krichevsky and Kosik, 2001; Kiebler and Bassell, 2006). The subcellular translation mediated by neuronal RNA granules regulates neural development, synapse formation, neuronal plasticity, and memory formation (Wang et al., 2010). Neuronal RNA granules are classified based on their localization, composition, and specialized functions in post-mitotic neurons (Mitsumori et al., 2017; Formicola et al., 2019). Indeed, neuronal RNA granules have greater diversity in RNA or protein composition than non-neuronal cells. For example, there are SGs, processing bodies (PBs), transport granules, and activity-dependent granules that are cytoplasmic RNA granules, which regulate mRNA metabolism (Hirokawa, 2006).

Among these several types of RNA granules, SGs are specialized cytoplasmic membraneless RNA granules, which are composed of SG core components including untranslated polyadenylated RNAs, RNA-binding proteins including poly-A binding protein (PABP), T-cell intracellular antigen1 (TIA-1), pre-initiation complex (PIC), and 40S ribosomal subunits, involved in pausing mRNA translation upon various cellular stresses (Decker and Parker, 2012). Although SG core components are common between subtypes of SGs, SG components are very heterogenous depending on cellular stress, or their subcellular localization (Aulas et al., 2017; Advani and Ivanov, 2020; Boncella et al., 2020). Several RNA-binding proteins, including SG nucleators or auxiliary factors, could contribute to the dynamic assembly or disassembly of SGs. They transiently and rapidly assemble with SG core components, other RNA binding proteins, or RNAs to form dynamic SGs, including shell-like layers of SGs via LLPS.

How is assembly of SGs regulated? Translational inhibition begins when stress is triggered. In general, the inhibition of translation associated with the assembly of SGs is tightly regulated by major signaling pathways. However, there are other signaling pathways involving protein synthesis and SG dynamics, depending on these types of stress in different cells: mammalian target of rapamycin (mTOR) activation status, eIF2α phosphorylation, and cap-binding eIF4F complex (Panas et al., 2016; Wolozin and Ivanov, 2019). In all cases, an incomplete formation of the pre-initiation complex for translation initiation leads to form the core structure of SGs and then assemble nucleate SGs by binding RNA binding proteins such as RAS GTP-activating protein-binding protein 1 (G3BP1) to mRNA or condensing each other. First, phosphorylation of eIF2α induces the arrest of translational initiation by impairing the formation of the ternary complex. Second, as the independent pathway of phosphorylation of eIF2α, mTOR inhibition also regulates the prevention of translational start by displacing eIF4A-eIF4G from the cap region of an mRNA by reducing the phosphorylation of eIF4E-binding protein (4E-BP). As the other pathway, the targeting of eIF4 cascade by tRNA-derived stress-induced RNAs (tiRNAs) or some drugs, such as silvestrol, affects the regulation of SG formation.

## Neuronal Transient Stress Granules as Dr. Jekyll

In highly polarized post-mitotic neurons compared to non-neuronal cells, normal functions of SGs are mostly unknown, although they generally contribute to pro-survival mechanisms upon various neuronal stresses (Shelkovnikova et al., 2017; Wolozin and Ivanov, 2019). However, many RNA-binding proteins related to SGs have been reported to regulate local protein synthesis in axon and dendrites to normalize neuronal signaling. Moreover, non-dividing neurons with highly polarized structures are believed to be more vulnerable to many acute cellular stresses, including oxidative, osmotic, proteotoxic, and chronic stresses, leading to an impairment of local translation and neuronal functions (Shelkovnikova et al., 2017; Advani and Ivanov, 2020).

Many RNA-binding proteins, such as FMRP and cytoplasmic polyadenylation element-binding protein (CPEB1) in dendrites and axon, regulate spatio-temporal regulation of translation of different target mRNAs. These translational controls could contribute to neural-specific functions, such as axonal or dendritic growth, synaptic development, and activity-dependent synaptic plasticity (Formicola et al., 2019). In particular, ataxin 2 (ATXN2) with IDR or CPEB1 induces granule formation and long-term memory (Sudhakaran et al., 2014; Sudhakaran and Ramaswami, 2017; Sahoo et al., 2018).

Very interestingly, many RNP complex proteins, including RNA-binding proteins, are localized to SGs upon cellular stresses. This might be due to the intrinsic reversible aggregation property of RNA-binding proteins. RNA-binding proteins generally have glycine-rich domains and RNA recognition motifs (RRM) (Wolozin, 2012). The glycine rich domain is known to be hydrophobic and involved in reversible aggregation and protein-protein interaction. The RRM domains are reported to different transcripts with broad specificity (López de Silanes et al., 2004, 2005; Kim et al., 2007; Liu et al., 2013).

The nucleocytoplasmic RNA binding proteins, such as Fused in Sarcoma (FUS) or Tar-DNA binding protein 43 kDa (TDP-43), mostly localize to the nucleus of healthy neurons without any stress. Once neurons are exposed to cellular stress, they rapidly localize to SGs. Translational repressor Pumilio 2 is known to be localized to dendrite and regulate assembly of SGs upon metabolic stress (Vessey et al., 2006). Regardless of their localization under normal conditions and cellular stress, these are rapidly translocated into the cytosol to form SGs, which must be tightly regulated by several factors, such as post-translational modification and RNA composition. The LLPS involved in the assembly and dynamics of SGs in the cytosol is mediated by heterotypic multivalent interactions, such as RNA-RNA or RNA-protein (Shorter, 2019; Babinchak and Surewicz, 2020). Under physiological conditions, LLPS promotes a high concentration of molecular interactions. For example, hnRNPA1, hnRNPA2B1, TIA-1, FUS, and TDP43, which are RNA-binding proteins and stress granule components undergo LLPS into protein-rich liquid droplets by IDR (Molliex et al., 2015). In some studies, IDR alone undergoes lipid droplets by phase separation *in vitro*. Furthermore, many studies suggest that RNA promotes phase separation. Another study showed that N-terminal fragment of hnRNPA1, which contains two RNA recognition motif (RRM) domains without IDR, undergoes LLPS in the presence of RNA (Molliex et al., 2015; Shin and Brangwynne, 2017).

Besides RNA-binding proteins, it has been reported that disease-associated protein, Tau or profilin1 or 2, is localized to SGs and regulates SG formation (Alkam et al., 2016; Vanderweyde et al., 2016). On the contrary, the interaction of Tau with TIA-1 as a stress granule component regulates Tau pathophysiology and toxicity, raising the possibility that normal stress granule components contribute to disease progression by promoting pathogenic aggregation (Vanderweyde et al., 2016).

A recent study reported that in the absence of cellular stress, G3BP1 as a stress granule component in axon, limits axonal mRNA translation and nerve regeneration, indicating that G3BP aggregates in SG-like structure in axon, regulating translational control (Sahoo et al., 2018).

To date, although neuronal subtypes of RNA granules have been extensively identified, their exact physiological roles in neurons remain unknown. Therefore, we need to identify and characterize the physiological role of subcellular-specific neuronal SGs in different types of neurons or different cellular contexts of glial cells. In addition, further mechanistic studies are needed to know different cellular stresses or disease states that could activate translocation of neuronal RNA-binding proteins in the subcellular compartment into the sites for the assembly of SGs. Moreover, there is a lack of evidence about the physiological roles of SGs *in vivo*.

## Abnormal Persistent SGs in Neurodegenerative Diseases: Mr. Hyde

**FIGURE 1:**
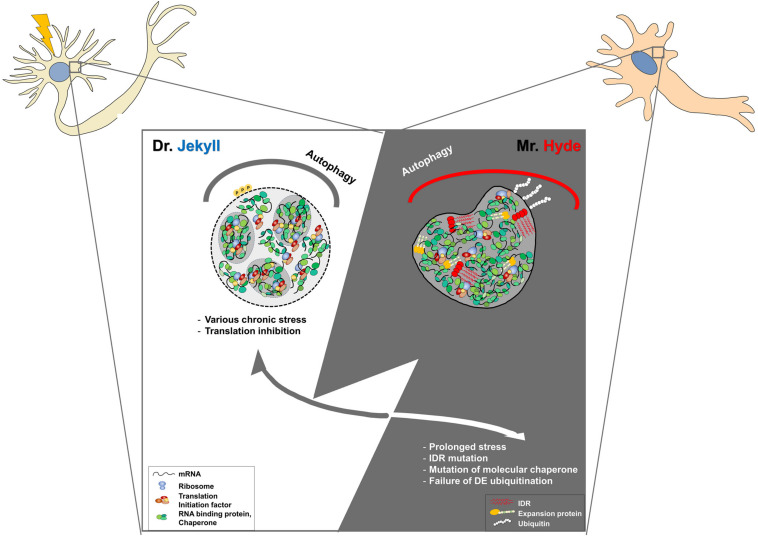
Development of physiological stress granules (SGs) into pathogenic SGs by chronic stress or genetic mutations in post-mitotic neurons. Dr. Jekyll: Stress granules are transiently formed to inhibit translational mRNA inhibition upon cellular stresses. Some SGs seem to be fused to other RNP granules, such as PBs, for their cooperation. When stress is removed, SGs are rapidly disassembled by molecular chaperons, VCP, the autophagy pathway, or proteasomal components. Mr. Hyde: However, the dynamics of SGs could be impaired by chronic stress during aging and genetic mutations in RNA-binding proteins, or the autophagy-associated pathway. In some cases, pathogenic SGs caused by genetic mutations in RNA binding proteins are formed without cellular stress and are persistent during aging, leading to the abnormal sequestration of cellular components and cellular toxicity. In the other cases, abnormal SGs caused by genetic mutations within SG disaggregate fail to be disassembled even after stress removal, leading to the accumulation of larger SGs. Dysregulation of the auto-lysosomal pathway could lead to the accumulation of SGs and impairment of the stress response in post-mitotic neurons. Therefore, targeting pathogenic SGs might be a promising therapeutic intervention.

Neuronal SGs are believed to have pro-survival roles upon various cellular stresses. Indeed, many mutations in several proteins related to SGs have been linked to various neurodegenerative diseases, including amyotrophic lateral sclerosis (Ryan and Fawzi, 2019; Wolozin and Ivanov, 2019; Advani and Ivanov, 2020). These mutations are found in aggregation-prone PLDs, LCDs, IDRs, and RNA-binding motifs within many RNA-binding proteins. LCDs or IDRs provide the conformational plasticity required for transient interactions between neighboring molecules associated with the dynamics of mRNP granules, leading to an impairment in their dynamics (Dobra et al., 2018). Accumulation of abnormal SGs might fuse each other to develop pathological aggregates found in several neurodegenerative diseases. Maturation of physiological SGs into pathogenic aggregates might be mainly caused by chronic stress, mutations in RNA-binding proteins, or RNA repeats associated with neurodegenerative diseases, with progressive impairment of their physiological disassembly or clearance (Figure 1).

Several neurodegenerative diseases are chronic and develop over many years. Their chronic characteristics indicate that cellular endogenous stress is much milder than the severe, acute exogenous stress used in laboratories. Prolonged and varied cellular stresses, such as oxidative stress, initially cause the formation of small or invisible SGs. They accumulate and fuse to each other to develop larger SGs over time, leading to visible insoluble aggregates in diseased brains. Therefore, pathogenic SG-associated aggregates are relatively larger and more insoluble in nature than physiological SGs.

Some mutant SG-associated proteins increase phase separation and self-propagation of SGs in a prion-like mechanism that is independent of stress, leading to pathogenic SGs associated with disease progression. This phenomenon might lead to aberrant phase transitions, to form abnormal insoluble SGs, removing SG dynamics. In the view of LLPS, disease-associated mutations are prevalently known to cause phase separation and alteration. For example, hnRNPA1 D262V, ALS-causing mutation, formed fibrils by LLPS (Molliex et al., 2015). Interestingly, several mutant proteins of TDP-43 and FUS are mis-localized to SGs from nucleus without cellular stress and further failed to the dynamic movement of SGs upon cellular stresses (Bentmann et al., 2013; Li et al., 2013; Ratti and Buratti, 2016). Indeed, alteration of LLPS by IDR mutation causes changes in properties of SG assembly or disassembly from liquid-like droplets to solid-like gels (Patel et al., 2015). Recent studies showed that the TDP43 liquid droplets in cytoplasm accumulate phosphorylated TDP43 and convert it into gel or solid state upon long lasting oxidative stress condition (Banani et al., 2017; Gasset-Rosa et al., 2019). PARylation also regulates stress granule dynamics, phase separation, and neurotoxicity of disease-related RNA-binding proteins, indicating that post-translational modification also affects LLPS (Duan et al., 2019).

Stress granules are considered to be a pathological seeding hub of RNA-binding proteins. This hypothesis was tested by Zhang et al. (2019) using chronic induction of optogenetic SGs in cellular model of FTD-ALS pathology. They showed that the persistent assembly of SGs is cytotoxic to cells and is accompanied by SG evolution to cytoplasmic inclusions that recapitulate ALS-FTD pathology (Zhang et al., 2019). In disease condition, SGs might be the essential link between environmental chronic stress and genetic mutations in several RNA-binding proteins (*HNRNPA2B1*, *EWS*, *MATR3 TIA1*, and *ATXN2)* and nucleotide-repeat expansion (FMRP or C9ORF72), as the accelerator of disease progression (Bassell and Warren, 2008; Lee et al., 2016; Boeynaems et al., 2017; Zhang et al., 2018; Wolozin and Ivanov, 2019). Regardless of their physiological functions, they are localized to SGs, impairing SG assembly, disassembly, and dynamics, leading to physiological SGs and pathogenic SG associated aggregates.

Finally, the impairment on SG disassembly or removal could cause an accumulation of abnormal SGs in post-mitotic neurons. In general, SGs are rapidly disassembled after the removal or degrading of cellular stress by autophagy (Buchan et al., 2013; Ryu et al., 2014). Indeed, autophagy-deficient cells accumulate SGs (Ryu et al., 2014). Moreover, gene mutations associated with autophagy have been identified in ALS patients (Li et al., 2013). Among these mutations, there are ubiquilin-2 (*UBQLN2*) or valosin-containing protein (*VCP*), that are SG disaggregates (Buchan et al., 2013; Ling et al., 2013; Wolozin and Ivanov, 2019). In addition, there are selective autophagy components, such as SQSTM1 (Rogov et al., 2014) (also called p62), serving as a ubiquitin-binding protein or as optineurin (OPTN) (Thomas et al., 2013). Heat-shock protein B8 (HSPB8) has been reported to regulate SG disassembly and the autophagic removal of misfolded protein (Ganassi et al., 2016). More recently, genetic mutations of TANK-binding kinase 1 (TBK1), which is a serine or threonine kinase that plays an essential role in regulating inflammatory responses and phosphorylation of OPTN, have been identified in FTD and ALS patients, supporting the possibility that links them in inflammation, neurodegeneration, and autophagy (Monahan et al., 2016).

The main question was whether the accumulation of abnormal SGs could contribute to disease progression or not. Our previous study using primary neuronal cell model expressing FUS R521C clearly showed that abnormal SG reduction by activating autophagy reduced neuronal toxicity caused by oxidative stress (Ryu et al., 2014). Furthermore, another study showed that drugs inducing autophagy rescued FUS stress in isogenic FUS-eGFP iPSC reporter lines (Marrone et al., 2018). Moreover, it has been reported that stress granule misprocessing *in vivo* revealed in a FUS knock-in ALS mouse model (Zhang et al., 2020). Therefore, cellular defects on SG disassembly or degradation contribute to disease progression, suggesting its detrimental roles in neuronal cell survival and functions.

Some evidences showed that SGs are seeds for pathogenic inclusions, associated with several neurodegenerative diseases contributing to disease progression, although this has no direct evidence *in vivo*. To date, there is no tool to specifically remove the pathogenic form of SGs *in vitro* and *in vivo*. Therefore, it is hard to conclude that abnormal SGs are the causing factors of cellular pathogenesis of diseases. In addition, although SG-associated inclusions are the hallmark of many neurodegenerative diseases, RNA-binding protein and diffused or small granules without forming larger SG aggregates, might promote disease pathology. To answer these questions, we need to identify and characterize SG subtypes in several neurodegenerative diseases and develop a specific tool that modifies or regulates specific types of SGs *in vitro* and *in vivo*.

## Discussion

In summary, highly ordered neuronal SGs and specific membraneless organelles are composed of polyadenylated mRNAs, RNA binding proteins, and PICs in axons, dendrites, and synapses of post-mitotic neurons. SGs are thought to be assembled or disassembled by the LLPS of several RNA-binding proteins. SGs have a protective role under stress, because they produce sensitive transcripts and contribute to signaling events, including anti-apoptotic signals, preferential translation of molecular chaperones, and other cytoprotective proteins (Buchan and Parker, 2009; Kim et al., 2013; Shelkovnikova et al., 2017). In neurons, the post-mitotic natures of SGs include highly polarized subcellular structure and dynamic signaling, making them more vulnerable to deficits in cellular stress responses, indicating that its dysregulation is strongly associated with neurodegeneration.

Recent accumulating studies indicate the existence of several subtypes of SGs, depending on cell and stress types. In the nervous system, there are neurons and glial cells, including astrocytes, microglia, and oligodendrocytes. The physiological roles of SGs in each cell type in the brain are largely unknown, although some SG markers are found in glial cells in diseased brains. Other major challenges are whether many types of SGs exhibit dynamic movement *in vivo* and the quantification method or tool for pathogenic inclusion that develop via SG-associated pathways. Further technical advances and studies are needed to identify and characterize neuronal SG subtypes.

Mutations in LCDs or PrLDs in RBDs or in genes in autophagy-associated pathways, together with chronic stress, affect SG dynamics and degradation, indicating that cellular deficits in stress responses lead to neurogenerative diseases, including ALS, FTD, and Alzheimer’s disease. Impairment of nuclear pore complexes, which mediate cytosolic shuttling of nuclear RBDs associated with several neurodegenerative diseases, may also be important. It is also an open question whether SGs are pre-existing forms that eventually evolve into the pathogenic protein inclusions, which are the hallmark of several neurodegenerative diseases. Since the hypothesis still lacks evidence *in vivo*, more studies are needed.

Despite these concerns, therapeutic approaches to regulate SGs are a good trial in treating several neurodegenerative diseases. If it is possible to reduce, disassemble, or clear abnormal SGs to inhibit their development into pathogenic aggregates, this might be a novel strategy to cure several neurodegenerative diseases associated with SGs (Ryan and Fawzi, 2019; Wang et al., 2020). However, there are several issues that must be considered by future studies to develop SG-targeting drugs. First, an *in vivo* animal model is needed to assess SGs in animals and the effects of drugs upon chronic stress. Second, since SGs are primarily protective for neuronal cell survival, targeting SGs by treatment of drugs also affect physiological survival. Therefore, we need to develop drugs that selectively target pathogenic SGs, and not the physiological ones. Finally, side effects are noted when SGs are targeted in treating neurodegenerative diseases.

## Author Contributions

JL conceived the manuscript. PJ and JL wrote the manuscript and designed the graph for the figure. Both authors contributed to the article and approved the submitted version.

## Conflict of Interest

The authors declare that the research was conducted in the absence of any commercial or financial relationships that could be construed as a potential conflict of interest.
